# Supplementary material for: The causal role of circulating inflammatory markers in osteoporosis: a bidirectional Mendelian randomized study
Source: Front Immunol. 2024 Jul 18;15:1412298. doi: 10.3389/fimmu.2024.1412298 (PMC11291241; doi:10.3389/fimmu.2024.1412298)
Supplement: Supplementary file 1 [file DataSheet_1.zip › supplementary material/OSTEOPOROSIS-CIM/GCST90274821.tsv.gz_forest.pdf]

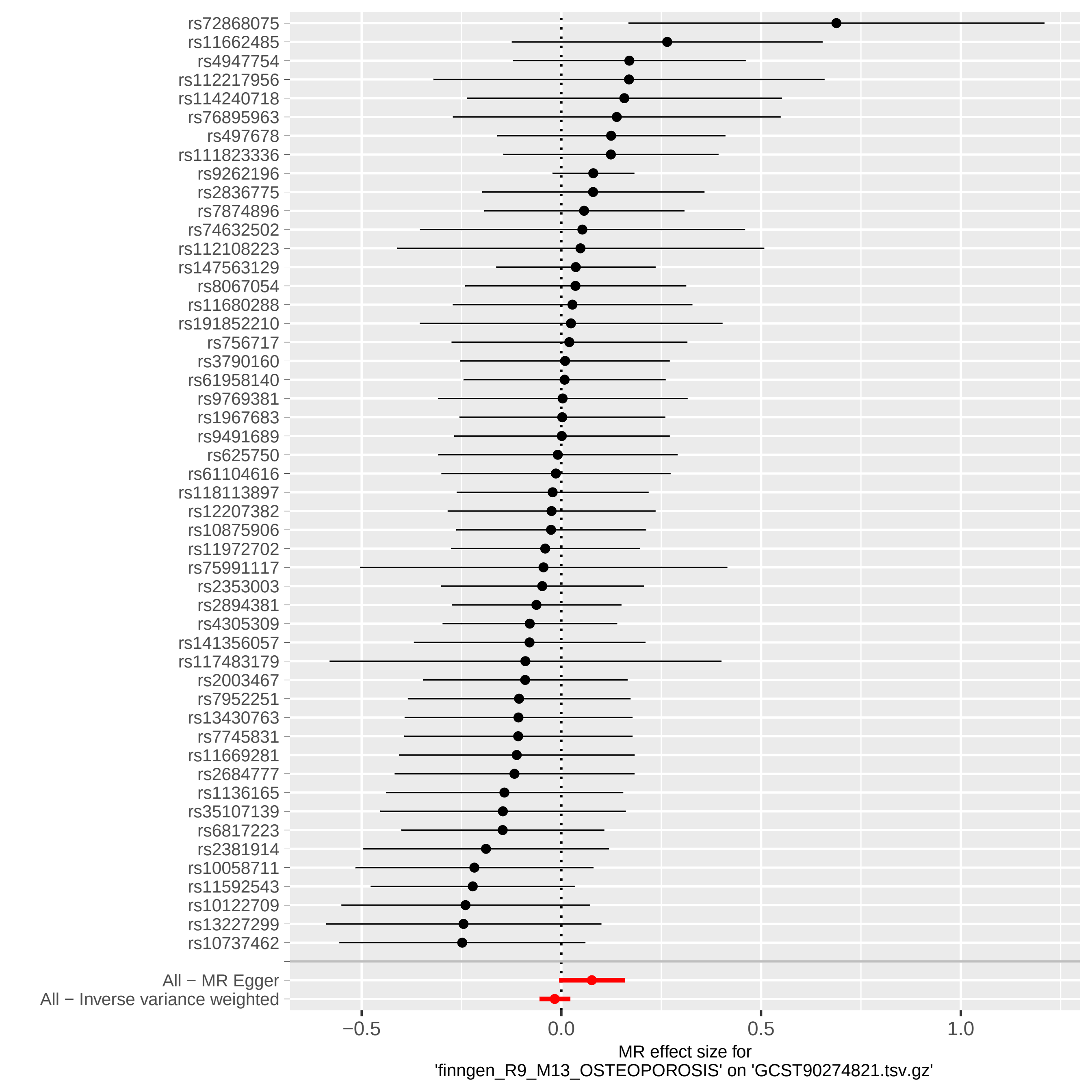
rs72868075
rs11662485
rs4947754
rs112217956
rs114240718
rs76895963
rs497678
rs111823336
rs9262196
rs2836775
rs7874896
rs74632502
rs112108223
rs147563129
rs8067054
rs11680288
rs191852210
rs756717
rs3790160
rs61958140
rs9769381
rs1967683
rs9491689
rs625750
rs61104616
rs118113897
rs12207382
rs10875906
rs11972702
rs75991117
rs2353003
rs2894381
rs4305309
rs141356057
rs117483179
rs2003467
rs7952251
rs13430763
rs7745831
rs11669281
rs2684777
rs1136165
rs35107139
rs6817223
rs2381914
rs10058711
rs11592543
rs10122709
rs13227299
rs10737462
All − MR Egger
All − Inverse variance weighted
−0.5
0.0
0.5
1.0
MR effect size for
'finngen_R9_M13_OSTEOPOROSIS' on 'GCST90274821.tsv.gz'
